# The relationship between physical activity and short video addiction among college students: mediating effects of self-control and social anxiety

**DOI:** 10.3389/fpsyg.2025.1640353

**Published:** 2025-09-18

**Authors:** Guoyu Wang, Kunbo Wu, Jiajie Gu, Zheng Zhang

**Affiliations:** ^1^School of Environmental Science and Engineering, Changzhou University, Changzhou, Jiangsu, China; ^2^School of Urban Construction, Changzhou University, Changzhou, Jiangsu, China

**Keywords:** physical activity, self-control, social anxiety, short video addiction, mediating effect, chain-mediated effects

## Abstract

**Introduction:**

Short video addiction has significantly altered college students’ learning, living, and entertainment habits, exerting a negative impact on their physical and mental health, which has drawn widespread attention from scholars.

**Methods:**

This study surveyed 650 college students using the Physical Activity Level Scale, Self-Control Scale, Adolescent Social Anxiety Scale, and Short Video Addiction Scale through the convenient sampling method to explore the mechanism of physical activity’s impact on short video addiction.

**Results:**

The results of the study indicate that: (1) physical activity has a significant and negative impact on short video addiction; (2) physical activity influences short video addiction through the mediating effect of self-control; (3) physical activity influences short video addiction through the mediating effect of social anxiety; (4) self-control and social anxiety play a chain mediating role in the impact of physical activity on short video addiction.

**Conclusion:**

These findings help researchers and educators better understand the underlying mechanisms of the relationship between physical activity and short video addiction, and provide practical and effective operational recommendations for the prevention and intervention of short video addiction among college students.

## 1 Introduction

In recent years, with the popularization of mobile Internet technology and the booming development of self-media technology, short videos have been rapidly emerged as an emerging Internet information dissemination carrier and communication method, greatly changing the way young people’s learning, life, and entertainment are conducted. Especially among college students, short videos are highly sought after for their short and concise, interesting content, and strong interactivity (Wu et al., 2021). However, this convenient way of information dissemination also brings new challenges. Excessive brushing of short videos by college students takes up a large amount of fragmented time, forming a black hole of time (Ye et al., 2025a,b), which leads to the pursuit of short-term pleasure gradually falling into a chronic or cyclical state of addiction (Wang, 2019), thus creating a continuous craving and dependence, i.e., the phenomenon of short video addiction (Levounis, 2024). Studies have shown that short video addiction is closely related to the reduced emotional regulation, distraction, and impaired social functioning of individuals (Montag et al., 2021). In short, this addictive behavior not only affects learning efficiency and time management but may also lead to physical and mental health problems such as distraction, decreased emotional regulation, decreased sleep quality, and even has a significant negative impact on social functioning (Ye et al., 2025a,b). Meanwhile, physical activity has been widely recognized as an effective form of psychological adjustment (Li, 2025; Shen et al., 2024). Studies have shown that moderate physical activity can improve psychological state, enhance self-control, and reduce anxiety (Martín-Rodríguez et al., 2024). By participating in physical activities, college students can not only improve physical fitness but also alleviate to some extent the negative emotions brought about by short video addiction (Qianqian et al., 2023). However, existing studies have not been completely clear about the intrinsic mechanisms and mediating roles regarding the relationship between physical activity and college students’ short video addiction. The present study aims to reveal how college students’ short video addiction behavior is affected by physical activity through the enhancement of self-control and regulation of social anxiety. By analyzing the dynamic relationship between them, theoretical support is provided for mental health interventions and the promotion of physical activity among college students, as well as practical experience for educators and college students in reducing short-video addiction and enhancing physical activity.

### 1.1 The relationship between physical activity and short video addiction

The relationship between physical activity as a positive health behavior and addictive behavior has been widely explored. According to self-control theory, it is suggested that reliance on immediate rewards is reduced through physical activity, which enhances an individual’s self-regulatory capacity and cognitive resource reserve (Baumeister et al., 2018), potentially inhibiting the development of addictive behaviors. In addition, stress coping theory states that negative emotions are alleviated by physical activity through the promotion of endorphin secretion and the lowering of cortisol levels (Childs and De Wit, 2014; Ye et al., 2023a), which in turn reduces alternative gratification behaviors of seeking transient pleasure through short videos (Liu et al., 2025). It has been found that the number of hours of physical activity per week among college students is significantly and negatively correlated with the length of short-video use (Yang et al., 2019), and that a lower tendency to become addicted is shown by individuals with higher levels of physical activity (Wu et al., 2025). Another experimental study indicates that better performance in terms of emotional stability and attentional control is demonstrated by students who participate in regular aerobic exercise, which directly reduces the dependent use of short videos (Zhou and Wang, 2022). Based on the above theoretical and empirical evidence, Hypothesis H1 is proposed in this paper.

*H1*: Physical activity has a significant negative effect on short video addiction among college students.

### 1.2 The mediating effect of self-control

Self-control, as an individual’s core psychological ability to regulate impulsive behaviors and achieve long-term goals, is considered to play a key mediating role in the dynamic interaction between healthy and addictive behaviors. According to the dual-system model of self-control, addictive behaviors are seen to arise from an imbalance between the impulsive and control systems (Duckworth et al., 2019). It is suggested that physical activity enhances an individual’s inhibitory control and delayed gratification ability by enhancing the cognitive control function of the brain region responsible for rational decision-making (Hillman et al., 2019), thereby indirectly reducing short video addictive behaviors through strengthening self-control. Meanwhile, ego depletion theory emphasizes that self-control resources are limited, and it is often found that short-video addiction is associated with excessive depletion of self-control resources required to resist immediate temptation (Hagger et al., 2010). Physical activity, as a regular, goal-directed activity, is believed to reduce addictive behaviors related to short-video use through the “self-control training effect” by increasing individuals’ resource reserve and recovery efficiency (Muraven, 2010). Longitudinal tracking studies have shown that individuals with lower self-control are more likely to rely on short videos to alleviate negative emotions under stress (Elhai et al., 2020), and it has been found that physical activity can block this reliance by increasing self-control efficacy in emotion regulation. Therefore, hypothesis H2 is proposed in this paper.

*H2*: Self-control mediates the relationship between physical activity and short video addiction among college students.

### 1.3 The mediating effect of social anxieties

Social anxiety is recognized as a prevalent psychological problem among the college student population, manifested by excessive worry about social situations, avoidance behaviors, and negative self-evaluations (Heimberg et al., 2010). It has been observed that with the popularity of short-video platforms, individuals with high social anxiety are more inclined to replace real socialization with virtual interactions in short videos, thus exacerbating the risk of addiction (Primack et al., 2017). According to social cognitive theory, it is suggested that social anxiety weakens an individual’s self-efficacy, leading to reliance on the feedback behavior of short videos to obtain transient psychological compensation (LaRose, 2009), thereby forming a vicious cycle of “avoidance-dependence-avoidance.” In addition, emotion regulation theory states that social anxiety is often accompanied by emotion regulation disorders, and it is argued that the immersive experience of short videos can quickly relieve anxiety, leading to addictive use (Gross, 2015). Some experimental studies have shown that significantly lower anxiety during social interactions is observed in individuals who participate in group exercise compared to sedentary individuals, and it has been found that their short video use duration decreases in tandem with addictive tendencies (Hunt et al., 2018; Nong et al., 2023). Based on this, hypothesis H3 is proposed in this paper.

*H3*: Social anxiety mediates the relationship between physical activity and short video addiction among college students.

### 1.4 Chain mediation effects of self-control and social anxieties

The theory of limited resources (Baumeister et al., 2018) states that self-control is dependent on limited psychological resources, and when a large amount of cognitive resources is consumed by social anxiety, the remaining resources are insufficient to inhibit immediate impulses, thus exacerbating the risk of addiction. Meanwhile, cognitive-behavioral theory emphasizes that social anxiety may motivate individuals to escape from real interactions through virtual social scenarios of short videos (Hofmann, 2007), and such alternative behaviors are predicated on a decline in self-control. It has been found by Kandola et al. (2019) that a negative correlation exists between college students’ level of social anxiety and their self-control, with those having low self-control being more inclined to alleviate their anxiety through low-threshold socialization methods such as short videos. Another study points out that social anxiety can weaken an individual’s emotion regulation efficacy, leading to accelerated depletion of self-control resources (Hofmann et al., 2012), which in turn increases the probability of addictive behaviors. Therefore, hypothesis H4 is proposed in this paper.

*H4*: Self-control and social anxiety play a chain mediating role between physical activity and short video addiction among college students.

### 1.5 The present study

**Figure 1:**
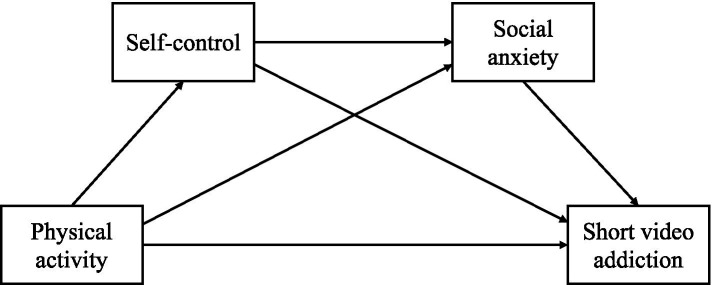
Hypothetical model among main variables.

In view of those analyses and discussions mentioned above, the effects of physical activity on college students’ short video addiction are investigated in this study, and the independent mediating effect of self-control and social anxiety as well as the chain mediating effects are also examined, so as to provide reference for the prevention and intervention of college students’ short video addiction. The study constructed a theoretical model between ‘physical activity,’ ‘self-control,’ ‘social anxiety,’ and ‘short video addiction’ (Figure 1), attempting to address three core issues:

(1) Whether physical activity reduces the risk of short video addiction by improving self-control ability;
(2) Whether social anxiety mediates the relationship between physical activity and short video addiction through emotion regulation;
(3) Whether there is a chain mediating effect between the two psychological mechanisms of self-control and social anxiety.

## 2 Research methodology

### 2.1 Participants

The questionnaire survey method is used to collect data in this study, with undergraduate students from a number of universities in Jiangsu Province being selected as the research object. The sampling method used by the research is convenience sampling, which is a non-probability sampling technique. The selection of samples is mainly based on accessibility or convenience. In order to encourage participants to participate in the study, individuals who are most easily accessible are selected as samples. Samples were taken from all grades and majors of the institutions where the researchers were located. From January to February 2025, a total of 668 questionnaires were distributed through the online questionnaire star, 688 were recovered, and 650 valid questionnaires were finally obtained by excluding invalid questionnaires, with an effective recovery rate of 94.5%. Therefore, the sample size for this study is 650. Among them, there are 195 female students (30.5%) and 453 male students (69.5%); 298 students (45.8%) in the first year of college, 88 students (13.5%) in the second year of college, 76 students (11.7%) in the third year of college, and 188 students (28.9%) in the fourth year of college; in terms of the average daily viewing of short videos, the number of those who watch short videos for less than an hour is 134 (20.6%), and the number of those who watch short videos for 1–2 h is 270 (20.5%). Two hours was 270 (41.5%), and more than 2 h was 246 (37.8%).

Before administering the questionnaire to the participants, we detailed the purpose and significance of the survey, and the data collected will only be used within the scope of the study. Participants were completely voluntary and anonymous throughout the study. Participants also have the right to withdraw from this study at any time.

### 2.2 Research tools

#### 2.2.1 Physical activity rating scale-3

The Physical Activity Rating Scale revised by Liang (1994) was used to investigate the three aspects of intensity, time, and frequency, with a 5-point Likert scale, and the amount of physical activity was calculated as “Exercise = Intensity × (Time-1) × Frequency.” The Cronbach′s *α* coefficient of the scale was 0.723.

#### 2.2.2 Self-control scale

The Self-Control Scale revised by Luo et al. (2021) was used, with a total of 7 entries and a 5-point Likert scale, with “1” to “5” denoting “not at all consistent” to “The higher the total score, the better the self-control ability, and the Cronbach’s *α* coefficient of this scale is 0.733.

#### 2.2.3 Social anxiety scale

The Social Anxiety Scale for Adolescents revised by Zhu et al. (2010) was used. The scale consists of 13 items, divided into three dimensions, and is scored using five points of Kert. From “1” to “5” means “not at all” to “completely conform,” with higher total scores representing higher levels of social anxiety. The Cronbach’s *α* coefficient of this scale is 0.891.

#### 2.2.4 Short video addiction scale

The short video addiction scale compiled by Qin (2020) was selected, with a total of 14 questions, consisting of four dimensions: loss of control, withdrawal, avoidance and inefficacy, and using a Likert 5-point scale, with “1” to “5” representing “The higher the score, the higher the individual’s tendency to become addicted to short videos. The Cronbach’s alpha coefficient for this scale is 0.907.

### 2.3 Statistical analyses

Descriptive statistics and Pearson correlation analysis were performed using SPSS 26.0. Since the data were obtained through questionnaires, to ensure the accuracy of the results, the covariance test was conducted using the Variance Inflation Factor (VIF) method (if the VIF is >10, it means that there is a serious covariance problem between the variables, and that the relevant variables need to be eliminated). For chained mediation effects analysis, the PROCESS macro program prepared by Hayes was used and the significance test for mediation effects was conducted using the bias-corrected percentile Bootstrap method. The mediating effect was considered statistically significant if the 99% confidence interval did not contain zero (Erceg-Hurn and Mirosevich, 2008).

## 3 Research results

### 3.1 Common method bias test

Principal component analysis using Harman’s one-factor test technique is used to test for common method bias (Podsakoff et al., 2003). It is shown that four factors have an eigen root greater than 1 and the variance explained by the first common factor is 32.9% < 40%. Therefore, no serious common method bias effect is found in this study.

### 3.2 Descriptive statistics and correlation analysis

The results of descriptive statistics and correlation analysis between the variables are shown in Table 1. The results show that these four variables are significantly correlated with each other: physical activity is significantly positively correlated with self-control ability, while it is significantly negatively correlated with social anxiety and short video addiction. Self-control was significantly negatively correlated with social anxiety and video addiction. Social anxiety is significantly positively correlated with short video addiction.

**Table 1:** Results of analysis of mean, standard deviation and correlation coefficient of the main study variables.

Variate	*M*	SD	Physical activity	Self-control	Social anxiety	Short video addiction
Physical activity	2.80	1.01				
Self-control	3.32	0.80	0.199**			
Social anxiety	3.16	0.89	−0.134**	−0.076**		
Short video addiction	2.73	0.89	−0.127**	−0.067**	0.499**	

**p < 0.01.

### 3.3 Validation factor analysis

Validated factor analysis was performed on the data in the scale using Amos 28.0. The results showed the following fit indices: the model indices for CMID and DF were 539.199 and 183, respectively, with smaller values indicating better fit. CMID/DF is 2.946, GFI is 0.925, AGFI is 0.905, CFI is 0.951, TLI is 0.943, RMSEA is 0.055, and SRMR is 0.081, all meeting the model fit criteria standards.

### 3.4 The test for mediation effects

**Figure 2:**
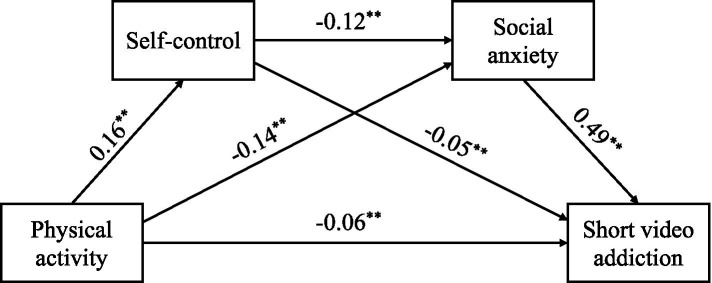
A chain-mediated effects model of self-control and social anxiety. *p < 0.05, **p < 0.01, ***p < 0.001.

Hierarchical regression analysis was performed using SPSS, and the path coefficients between variables are shown in Figure 1. Physical activity had a significant negative predictive effect on short video addiction (*β* = −0.06, *p* < 0.01); at the same time, there was also a significant direct effect of physical activity on self-control (*β* = 0.16, *p* < 0.001) and social anxiety (*β* = −0.14, *p* < 0.001); self control had a significant negative predictive effect on short-video addiction (*β* = −0.05, *p* < 0.0); social anxiety had a significant positive predictive effect on short-video addiction (*β* = 0.49, *p* < 0.001); and between the two mediating variables, i.e., self-control had a significant negative predictive effect on social anxiety (*β* = −0.12, *p* < 0.01) (Figure 2).

The study used Amos 28.0 to analyze the parameters of the structural equation model and chose the Bootstrap method to test the significance of the chain mediation by performing 5,000 replicates and calculating the 95% confidence interval for the mediation effect, which if there are no zeros in the interval, then it represents a significant mediation effect of the mediating variable, and if the interval contains zeros, then it represents a non-significant mediation effect of the mediating variable (Ye and Wen, 2013). The results of the mediating effect analysis showed (as shown in Table 2) that the path effect value of physical activity → self-control → college students’ short-video addiction was −0.008 (95% CI [−0.023, −0.006]), with an effect size of 5.35%, which verified the partial mediating role of self-control. This result is consistent with self-determination theory: physical activity enhances self-control resources by increasing an individual’s sense of autonomy and competence (Tangney et al., 2018), which in turn inhibits impulsive use of short videos. The path effect value of physical activity → social anxiety → short video addiction among college students was −0.067 (95% CI [−0.030, −0.011]), with an effect size of 45.78%. The results indicated that physical activity significantly reduced college students’ dependence behavior on short videos by lowering the level of social anxiety. Social anxiety, as a key variable in stress coping theory (Caplan, 2003), effectively reduces individuals’ dependence on virtual social environments, thus breaking the vicious cycle of “anxiety-avoidance-addiction.” The effect value of physical activity → self-control → social anxiety → college students’ short video addiction chain path was −0.009 (95% CI [−0.021, −0.001]), and the effect size accounted for 6.24%. The significance suggests a progressive relationship between self-control and social anxiety: physical activity not only directly enhances self-control, but also may further alleviate social anxiety and ultimately reduce the risk of addiction by enhancing the reserve of psychological resources (Brand et al., 2016).

**Table 2:** Chained mediation effect test and analysis results.

Effect path	Effect value	Boot LL CI	Boot UL CI	Magnitude of effect
Total effect	−0.146	−0.180	−0.044	100.00%
Direct effect	−0.062	−0.123	−0.001	42.63%
Aggregate intermediary effect	−0.084	−0.093	−0.010	57.37%
Physical activity = > Self-control = > Short video addiction	−0.008	−0.023	−0.006	5.35%
Physical activity = > Social anxiety = > Short video addiction	−0.067	−0.030	−0.011	45.78%
Physical activity = > Self-control = > Social anxiety = > Short video addiction	−0.009	−0.021	−0.001	6.24%

## 4 Discussion

### 4.1 Analysis of the chain mediating mechanism of physical activity and short video addiction

Firstly, there is a significant negative correlation between physical activity and short video addiction among college students, validating hypothesis H1. Specifically, physical activity is shown to help divert students’ attention away from short video platforms by providing them with opportunities for physical activity (Yang et al., 2022), thereby reducing the likelihood of addiction. Regular physical activity is not only beneficial for physical health but also promotes mental health and alleviates the negative effects of excessive short video use (Mahindru et al., 2023). The study finds that the amount of time spent on physical activity per week is significantly negatively correlated with the duration of short video use, and individuals with higher levels of physical activity exhibit lower addiction tendencies (Wu et al., 2025; Yang et al., 2021). Additionally, students who engage in regular aerobic exercise demonstrate superior emotional stability and attention control, which directly reduces dependent use of short videos (Zhou and Wang, 2022). These findings highlight the potential of physical activity as an effective psychological adjustment method in preventing and intervening in short video addiction. Therefore, college students are encouraged to actively participate in physical activities, which not only improves their physical fitness but also enhances their mental health, thereby reducing the risk of short video addiction.

Secondly, self-control, as a core psychological ability for individuals to regulate impulsive behavior and achieve long-term goals, plays a key mediating role in the dynamic interaction between healthy behavior and addictive behavior. The results of this study indicate that self-control exerts a significant positive mediating effect between physical activity and college students’ short video addiction, thereby validating Hypothesis H2. Based on self-determination theory (Chen et al., 2022), physical activity has a positive effect on enhancing individuals’ self-control abilities. High levels of self-control enable college students to better resist the temptation of immediate gratification (Watson and Milfont, 2017), such as reducing prolonged short video viewing behavior (Ye et al., 2024). Physical activity enhances cognitive control functions in brain regions responsible for rational decision-making, thereby improving individuals’ inhibitory control and delayed gratification abilities, and indirectly reducing short video addiction behavior (Wu et al., 2022). Additionally, self-depletion theory suggests that self-control resources are limited (Baumeister et al., 2024), and short video addiction is often associated with excessive depletion of self-control resources to resist immediate temptations. Physical activity, as a regular, goal-oriented activity, may enhance individuals’ resource reserves and recovery efficiency through the “self-control training effect,” thereby reducing addictive behavior related to short video use (Jianfeng et al., 2024). Therefore, by participating in physical activities, college students can enhance their self-management abilities, thereby more effectively controlling their short video usage habits and reducing the risk of addiction.

Thirdly, social anxiety is a common psychological issue among college students, manifested as excessive worry about social environments, avoidance behaviors, and negative self-evaluation. Research indicates that with the widespread adoption of short video platforms, individuals with high levels of social anxiety are more likely to replace real-life social interactions with virtual interactions on short video platforms, thereby exacerbating the risk of addiction (Reer et al., 2022). According to social cognitive theory (Luszczynska and Schwarzer, 2015), social anxiety may weaken an individual’s sense of self-efficacy, leading them to rely on feedback from short videos to obtain temporary psychological compensation, thereby forming a vicious cycle of “avoidance-dependence-avoidance.” Additionally, emotion regulation theory suggests that social anxiety often co-occurs with emotion regulation disorders (Chen, 2016), and the immersive experience of short videos can quickly alleviate anxiety, leading to addictive use. This study further found that social anxiety plays a significant negative mediating role between physical activity and short video addiction among college students, validating hypothesis H3. Physical activities provide students with opportunities for offline social interaction, promote the development of interpersonal skills, and reduce feelings of loneliness and social isolation. This emotional improvement reduces students’ reliance on virtual social platforms for psychological comfort, thereby decreasing short video usage frequency and addiction risk. Thus, physical activity is not only an effective means of improving physical fitness but also an important strategy for alleviating social anxiety and reducing short video addiction.

Finally, this study also revealed the chained mediating role of self-control and social anxiety in the relationship between physical activity and college students’ short video addiction, thereby validating Hypothesis H4. Self-control and social anxiety, as chained mediating variables, played a significant role in the relationship between physical activity and college students’ short video addiction. Physical activity enhances students’ self-control, enabling them to resist the temptation of short videos more effectively (Yu and Mu, 2024). Additionally, physical activities help students overcome social anxiety, reducing their need to seek solace in the virtual world (Ke et al., 2024). This finding highlights the potential mechanisms through which physical activity can improve mental health and reduce short video addiction behavior, thereby refining the chained mediation effect model. Specifically, the path effect value for the path from physical activity to self-control to college students’ short video addiction is −0.008 (95% CI [−0.023, −0.006]), with an effect size of 5.35%; the path effect value for the chained pathway from physical activity → self-control → short video addiction among college students is −0.067 (95% CI [−0.030, −0.011]), with an effect size of 45.78%; the chained path effect value from physical activity → self-control → social anxiety → short video addiction among college students was −0.009 (95% CI [−0.021, −0.001]), with an effect size of 6.24%. These results indicate that physical activity not only directly enhances self-control but may also alleviate social anxiety by strengthening psychological resources, ultimately reducing the risk of addiction. This finding provides theoretical support for developing effective intervention strategies.

### 4.2 Theoretical value

This study investigates the relationship between physical activity, self-control, social anxiety, and short video addiction, verifying the chain-mediated role of self-control and social anxiety in this context. This not only enriches existing theoretical frameworks, such as self-determination theory and stress coping theory, but also provides new insights into understanding addictive behavior. Specifically, we found that physical activity not only directly reduces short video addiction but also indirectly influences addictive behavior by enhancing individuals’ self-control abilities and alleviating social anxiety. This finding further supports the theory of limited self-control resources and reveals the mechanism through which physical activity, as an important means of regulating mental health, plays a role in preventing addictive behavior. Additionally, the results of this study provide a theoretical foundation for future research, particularly in exploring other potential mediating variables and their interactions.

### 4.3 Research implications

#### 4.3.1 Promoting mental health among college students

This study highlights the significant role of physical activity in enhancing college students’ self-control abilities and alleviating social anxiety, thereby reducing short video addiction. For college students, this finding has important practical implications. First, college life is filled with various challenges and pressures, from academic burdens to interpersonal relationships, all of which can have a negative impact on students’ mental health (Monserrat-Hernández et al., 2023). By participating in physical activities, students can not only improve their physical fitness but also effectively alleviate psychological stress and enhance emotional stability. For example, regular aerobic exercise can promote the secretion of endorphins, helping individuals better cope with daily stress and thereby improving overall mental health (Giandonato et al., 2021). Additionally, sports activities provide students with opportunities to interact with others, fostering teamwork and social skills—particularly important for those with social anxiety issues (Li et al., 2024; Ye et al., 2022). By increasing offline social interactions, students can gradually overcome their reliance on virtual social platforms and build more authentic and healthy social networks. Therefore, encouraging college students to actively engage in physical activity not only helps prevent short video addiction but also comprehensively improves their mental health, enabling them to better adapt to the various challenges of university life.

#### 4.3.2 Provide effective intervention strategies

The findings of this study provide a solid theoretical foundation and empirical support for developing effective intervention measures. Specifically, schools can promote physical activity through various channels to reduce short video addiction. On the one hand, schools can organize diverse sports events and activities to attract students with different interests and hobbies to participate. This not only enhances students’ physical fitness but also improves their mental health. For example, schools can establish a fixed weekly sports activity day to encourage students to step out of the classroom and engage in various forms of physical activity. On the other hand, schools can also organize specialized lectures or workshops, inviting psychology experts to explain how to cultivate good self-control habits and share specific techniques and methods. These lectures can help students understand the importance of self-control and master practical strategies to enhance their self-management abilities. At the same time, parents should also take on their corresponding responsibilities, creating a healthy living environment at home, guiding children to reduce their exposure to electronic devices, and encouraging them to participate in family activities and social practices (Ye et al., 2023b). For example, parents can establish clear family rules, such as limiting daily screen time or encouraging family members to engage in outdoor activities together, to promote children’s holistic development. Through collaborative efforts, we can create a healthier digital living environment for young people and help them avoid the harms of short video addiction.

#### 4.3.3 Promoting the establishment of social support systems

This study emphasizes the importance of multi-party collaboration, including schools, families, and society, to jointly assume responsibility for creating a healthier digital living environment for young people. Society should provide psychological counseling and guidance services to help students in need overcome difficulties, build confidence, and enhance self-identity. For example, schools can establish counseling centers to regularly provide mental health assessments and counseling services for students, helping them identify and address potential psychological issues. In addition, communities can also organize various mental health education activities to raise public awareness of the importance of mental health. Through such efforts, we can create a supportive system conducive to the healthy development of adolescents, helping them avoid the harms of short video addiction. At the same time, all sectors of society should strengthen regulation of short video content to ensure it aligns with the developmental needs and direction of adolescents. For example, governments can enact relevant policies and regulations to standardize content review mechanisms on short video platforms, reducing the impact of harmful information on adolescents. Furthermore, media and technology companies should also assume social responsibility by developing more applications and services that benefit the physical and mental development of adolescents. Through collaborative efforts, we can collectively create a healthy and positive digital living environment for adolescents, supporting their healthy growth.

### 4.4 Insufficient research and prospects

The findings of this study offer important insights into preventing short video addiction. First, encourage college students to actively participate in physical activity. To effectively reduce short video addiction, schools can organize diverse sports events and activities to attract students with different interests, which not only enhances physical fitness but also alleviates academic stress and improves mental health. Second, enhance college students’ self-control through multiple channels. On one hand, schools can host specialized lectures or workshops, inviting psychology experts to explain how to cultivate good self-control habits and share specific techniques and methods. On the other hand, parents should also take on their respective responsibilities, creating a healthy living environment at home, guiding individuals to reduce their exposure to electronic devices, and encouraging them to participate more in family activities and social practices.

This study also has the following limitations. First, this study used a cross-sectional design, which limits the ability to draw causal inferences. Cross-sectional data makes it difficult to completely rule out reverse causality or interference from third-party variables. Second, the sample characteristics have certain limitations. Considering that students of different grades and genders may have differences in lifestyle and psychological state, future studies can further subdivide the sample population to analyze the specific performance of students in each grade. Third, technological developments have opened up new possibilities for research. The widespread use of wearable devices and mobile health apps not only enables the collection of more objective data but also allows for dynamic monitoring, thereby providing a better understanding of the specific mechanisms through which physical activity influences mental health.

Future research could also explore other potential mediating variables and their interactions, such as personality traits and family background. With the advancement of technology, the widespread use of wearable devices and mobile health apps has opened up new possibilities. These technologies not only provide more objective data but also enable dynamic monitoring, thereby helping us better understand the specific mechanisms through which physical activity influences mental health. Additionally, conducting in-depth studies on specific populations (such as students with a high risk of addiction) can help develop more targeted preventive measures. By continuously deepening our understanding of this complex network of relationships, we can create a healthier digital living environment for young people.

## 5 Conclusion

The impact of physical activity on short video addiction and the chained mediating effects of self-control and social anxiety in this process are investigated in this study. The findings indicate that physical activity is negatively correlated with short video addiction among college students. The more time spent on physical activity, the lower the level of short video addiction. The relationship between physical activity and short video addiction among college students is partially moderated by self-control and social anxiety. Furthermore, the effects of self-control and social anxiety are closely interrelated, with both exhibiting a sequential mediating effect in the process by which physical activity influences short video addiction.

## Data Availability

The raw data supporting the conclusions of this article will be made available by the authors, without undue reservation.

## References

[ref1] Baumeister R. F. André N. Southwick D. A. Tice D. M. (2024). Self-control and limited willpower: current status of ego depletion theory and research. Curr. Opin. Psychol. 60:101882. doi: 10.1016/j.copsyc.2024.101882, PMID: 39278166

[ref2] Baumeister R. F. Bratslavsky E. Muraven M. Tice D. M. (2018). Ego depletion: is the active self a limited resource? J. Pers. Soc. Psychol. 74, 1252–1265. doi: 10.1037/0022-3514.74.5.1252 9599441

[ref3] Brand M. Young K. S. Laier C. Wölfling K. Potenza M. N. (2016). Integrating psychological and neurobiological considerations regarding the development and maintenance of specific internet-use disorders: an interaction of person-affect-cognition-execution (I-PACE) model. Neurosci. Biobehav. Rev. 71, 252–266. doi: 10.1016/j.neubiorev.2016.08.033, PMID: 27590829

[ref4] Caplan S. E. (2003). Preference for online social interaction: a theory of problematic internet use and psychosocial well-being. Commun. Res. 30, 625–648. doi: 10.1177/0093650203257842

[ref5] Chen H. (2016). A theoretic review of emotion regulation. Open J. Soc. Sci. 4, 147–153. doi: 10.4236/jss.2016.42020

[ref6] Chen S. Wang Q. Wang X. Huang L. Zhang D. Shi B. (2022). Self-determination in physical exercise predicts creative personality of college students: the moderating role of positive affect. Front. Sports Active Living 4:926243. doi: 10.3389/fspor.2022.926243, PMID: 35899140 PMC9309356

[ref7] Childs E. De Wit H. (2014). Regular exercise is associated with emotional resilience to acute stress in healthy adults. Front. Physiol. 5:161. doi: 10.3389/fphys.2014.00161, PMID: 24822048 PMC4013452

[ref8] Duckworth A. L. Taxer J. L. Eskreis-Winkler L. Galla B. M. Gross J. J. (2019). Self-control and academic achievement. Annu. Rev. Psychol. 70, 373–399. doi: 10.1146/annurev-psych-010418-103230, PMID: 30609915

[ref9] Elhai J. D. Yang H. Montag C. (2020). Fear of missing out (FOMO): overview, theoretical underpinnings, and literature review on relations with severity of negative affectivity and problematic technology use. Braz. J. Psychiatry 43, 203–209. doi: 10.1590/1516-4446-2020-0870 PMC8023172 32401865

[ref10] Erceg-Hurn D. M. Mirosevich V. M. (2008). Modern robust statistical methods: an easy way to maximize the accuracy and power of your research. Am. Psychol. 63, 591–601. doi: 10.1037/0003-066X.63.7.591, PMID: 18855490

[ref11] Giandonato J. A. Tringali V. M. Thoms R. C. (2021). Improving mental health through physical activity: a narrative literature review. Phys. Act. Health 5, 146–153. doi: 10.5334/paah.108

[ref12] Gross J. J. (2015). Emotion regulation: current status and future prospects. Psychol. Inq. 26, 1–26. doi: 10.1080/1047840X.2014.940781

[ref13] Hagger M. S. Wood C. Stiff C. Chatzisarantis N. L. (2010). Ego depletion and the strength model of self-control: a meta-analysis. Psychol. Bull. 136, 495–525. doi: 10.1037/a0019486, PMID: 20565167

[ref14] Heimberg R. G. Brozovich F. A. Rapee R. M. (2010). A cognitive behavioral model of social anxiety disorder: update and extension. Social anxiety. Academic Press, 395–422. doi: 10.1016/B978-0-12-375096-9.00015-8

[ref15] Hillman C. H. Logan N. E. Shigeta T. T. (2019). A review of acute physical activity effects on brain and cognition in children. Transl. J. Am. Coll. Sports Med. 4, 132–136. doi: 10.1249/TJX.0000000000000101

[ref16] Hofmann S. G. (2007). Cognitive factors that maintain social anxiety disorder: a comprehensive model and its treatment implications. Cogn. Behav. Ther. 36, 193–209. doi: 10.1080/16506070701421313, PMID: 18049945 PMC2151931

[ref17] Hofmann W. Baumeister R. F. Förster G. Vohs K. D. (2012). Everyday temptations: an experience sampling study of desire, conflict, and self-control. J. Pers. Soc. Psychol. 102, 1318–1335. doi: 10.1037/a0026545, PMID: 22149456

[ref18] Hunt M. G. Marx R. Lipson C. Young J. (2018). No more FOMO: limiting social media decreases loneliness and depression. J. Soc. Clin. Psychol. 37, 751–768. doi: 10.1521/jscp.2018.37.10.751

[ref19] Jianfeng H. Xian Z. Zexiu A. (2024). Effects of physical exercise on adolescent short video addiction: a moderated mediation model. Heliyon 10:e29466. doi: 10.1016/j.heliyon.2024.e29466, PMID: 38638962 PMC11024624

[ref20] Kandola A. Ashdown-Franks G. Hendrikse J. Sabiston C. M. Stubbs B. (2019). Physical activity and depression: towards understanding the antidepressant mechanisms of physical activity. Neurosci. Biobehav. Rev. 107, 525–539. doi: 10.1016/j.neubiorev.2019.09.040, PMID: 31586447

[ref21] Ke Y. Liu X. Xu X. He B. Wang J. Zuo L.. (2024). Self-esteem mediates the relationship between physical activity and smartphone addiction of Chinese college students: a cross-sectional study. Front. Psychol. 14:1256743. doi: 10.3389/fpsyg.2023.1256743, PMID: 38250119 PMC10797096

[ref22] LaRose R. (2009). Social cognitive theories of media selection. Media choice. Routledge. 24–45.

[ref23] Levounis P. (2024). Technological addictions: the new frontier in addiction psychiatry. Eur. Psychiatry 67:S410. doi: 10.1192/j.eurpsy.2024.845

[ref24] Li L. (2025). The influence of physical exercise on the mental and emotional regulation ability of college students. Schizophr. Bull. 51:S23. doi: 10.1093/schbul/sbaf007.043

[ref25] Li Z. Li J. Kong J. Li Z. Wang R. Jiang F. (2024). Adolescent mental health interventions: a narrative review of the positive effects of physical activity and implementation strategies. Front. Psychol. 15:1433698. doi: 10.3389/fpsyg.2024.1433698, PMID: 38993342 PMC11236730

[ref26] Liang D. (1994). Stress levels among college students and their relationship to physical exercise. Chin. Ment. Health J. 8, 5–6.

[ref27] Liu Y. Huang Y. Wen L. Chen P. Zhang S. (2025). Temporal focus, dual-system self-control, and college students' short-video addiction: a variable-centered and person-centered approach. Front. Psychol. 16:1538948. doi: 10.3389/fpsyg.2025.1538948, PMID: 40196206 PMC11973371

[ref28] Luo T. Cheng L. Qin L. Xiao S. (2021). Validity and reliability testing of the Chinese version of the brief self-control scale. Chin. J. Clin. Psychol. 29, 83–86. doi: 10.16128/j.cnki.1005-3611.2021.01.017

[ref29] Luszczynska A. Schwarzer R. (2015). Social cognitive theory. Fac Health Sci Publ 2015, 225–251.

[ref30] Mahindru A. Patil P. Agrawal V. (2023). Role of physical activity on mental health and well-being: a review. Cureus 15:3475. doi: 10.7759/cureus.33475, PMID: 36756008 PMC9902068

[ref31] Martín-Rodríguez A. Gostian-Ropotin L. A. Beltrán-Velasco A. I. Belando-Pedreño N. Simón J. A. López-Mora C.. (2024). Sporting mind: the interplay of physical activity and psychological health. Sports 12:37. doi: 10.3390/sports12010037, PMID: 38275986 PMC10819297

[ref32] Monserrat-Hernández M. Checa-Olmos J. C. Arjona-Garrido Á. López-Liria R. Rocamora-Pérez P. (2023). Academic stress in university students: the role of physical exercise and nutrition. Healthcare 11:2401. doi: 10.3390/healthcare11172401, PMID: 37685435 PMC10486982

[ref33] Montag C. Yang H. Elhai J. D. (2021). On the psychology of TikTok use: a first glimpse from empirical findings. Front. Public Health 9:641673. doi: 10.3389/fpubh.2021.641673, PMID: 33816425 PMC8010681

[ref34] Muraven M. (2010). Building self-control strength: practicing self-control leads to improved self-control performance. J. Exp. Soc. Psychol. 46, 465–468. doi: 10.1016/j.jesp.2009.12.011, PMID: 20401323 PMC2855143

[ref35] Nong W. He Z. Ye J.-H. Wu Y.-F. Wu Y.-T. Ye J.-N.. (2023). The relationship between short video flow, addiction, serendipity, and achievement motivation among Chinese vocational school students: the post-epidemic era context. Healthcare 11:462. doi: 10.3390/healthcare11040462, PMID: 36832995 PMC9957412

[ref36] Podsakoff P. M. MacKenzie S. B. Lee J.-Y. Podsakoff N. P. (2003). Common method biases in behavioral research: a critical review of the literature and recommended remedies. J. Appl. Psychol. 88, 879–903. doi: 10.1037/0021-9010.88.5.879, PMID: 14516251

[ref37] Primack B. A. Shensa A. Sidani J. E. Whaite E. O. Lin L. Rosen D.. (2017). Social media use and perceived social isolation among young adults in the US. Am. J. Prev. Med. 53, 1–8. doi: 10.1016/j.amepre.2017.01.010, PMID: 28279545 PMC5722463

[ref38] Qianqian L. Yan S. Longlong W. Peng W. Sang X. (2023). Influence of sports elective courses on college students' physical health. Rev. Bras. Med. Esporte 29:e2022_0790. doi: 10.1590/1517-8692202329012022_0790, PMID: 39245730

[ref39] Qin H. (2020). Research on the influence mechanism and intervention countermeasures of short video addiction among college students. Jiangxi Norm. Univ. Sci. Technol. 6, 15–17. doi: 10.27751/d.cnki.gjxkj.2020.000299

[ref40] Reer F. Wehden L.-O. Janzik R. Tang W. Y. Quandt T. (2022). Virtual reality technology and game enjoyment: the contributions of natural mapping and need satisfaction. Comput. Human Behav. 132:107242. doi: 10.1016/j.chb.2022.107242

[ref41] Shen Q. Wang S. Liu Y. Wang Z. Bai C. Zhang T. (2024). The chain mediating effect of psychological inflexibility and stress between physical exercise and adolescent insomnia. Sci. Rep. 14:24348. doi: 10.1038/s41598-024-75919-8, PMID: 39420219 PMC11486977

[ref42] Tangney J. P. Boone A. L. Baumeister R. F. (2018). High self-control predicts good adjustment, less pathology, better grades, and interpersonal success. Self-regulation and self-control, 173–212. doi: 10.1111/j.0022-3506.2004.00263.x 15016066

[ref43] Wang W.-C. (2019). Exploring the relationship among free-time management, leisure boredom, and internet addiction in undergraduates in Taiwan. Psychol. Rep. 122, 1651–1665. doi: 10.1177/0033294118789034, PMID: 30071775

[ref44] Watson S. J. Milfont T. L. (2017). A short-term longitudinal examination of the associations between self-control, delay of gratification and temporal considerations. Pers. Individ. Differ. 106, 57–60. doi: 10.1016/j.paid.2016.10.023

[ref45] Wu S. Li D. Wang Y. Dai F. Hong Y. (2021). Study on the acceptance behavior o f college students to the ways of acquiring short video tourism information based o n the extended UTAUT model. Converter 7, 604–618.

[ref46] Wu J. Shao Y. Zang W. (2025). The impact of physical exercise on adolescents’ mobile phone dependency: the serial mediating role of self-esteem and depression. Front. Psychol. 16:1471657. doi: 10.3389/fpsyg.2025.1471657, PMID: 39995430 PMC11847896

[ref47] Wu J. Xiao W. Yip J. Peng L. Zheng K. Takyi Bentil O.. (2022). Effects of exercise on neural changes in inhibitory control: an ALE meta-analysis of fMRI studies. Front. Hum. Neurosci. 16:891095. doi: 10.3389/fnhum.2022.891095, PMID: 35814955 PMC9265250

[ref48] Yang G. Li Y. Liu S. Liu C. Jia C. Wang S. (2021). Physical activity influences the mobile phone addiction among Chinese undergraduates: the moderating effect of exercise type. J. Behav. Addict. 10, 799–810. doi: 10.1556/2006.2021.00059, PMID: 34546969 PMC8997213

[ref49] Yang G. Shangguan R. Ke Y. Wang S. (2022). The influence of acute aerobic exercise on craving degree for university students with mobile phone dependency: a randomized controlled trial. Int. J. Environ. Res. Public Health 19:8983. doi: 10.3390/ijerph19158983, PMID: 35897357 PMC9331807

[ref50] Yang G. Tan G.-x. Li Y.-x. Liu H.-y. Wang S.-t. (2019). Physical exercise decreases the mobile phone dependence of university students in China: the mediating role of self-control. Int. J. Environ. Res. Public Health 16:4098. doi: 10.3390/ijerph16214098, PMID: 31652978 PMC6862431

[ref51] Ye J.-H. Cui Y. Wang L. Ye J.-N. (2024). The relationships between the short video addiction, self-regulated learning, and learning well-being of Chinese undergraduate students. Int. J. Ment. Health Promot. 26, 805–815. doi: 10.32604/ijmhp.2024.055814

[ref52] Ye J.-H. He Z. Yang X. Lee Y.-S. Nong W. Ye J.-N.. (2023a). Predicting the learning avoidance motivation, learning commitment, and silent classroom behavior of Chinese vocational college students caused by short video addiction. Healthcare 11:985. doi: 10.3390/healthcare11070985, PMID: 37046912 PMC10094292

[ref53] Ye J.-H. Wang Y. Nong W. Ye J.-N. Cui Y. (2025a). Relationship between TikTok (Douyin) addiction and social and emotional learning: evidence from a survey of Chinese vocational college students. Int. J. Ment. Health Promot. 27:7. doi: 10.32604/ijmhp.2025.066326

[ref54] Ye B. Wen Z. (2013). A discussion on testing methods for mediated moderation models: discrimination and integration. Acta Psychol. Sin. 45, 1050–1060. doi: 10.3724/sp.j.1041.2013.01050

[ref55] Ye J.-H. Wu Y.-F. Nong W. Wu Y.-T. Ye J.-N. Sun Y. (2023b). The association of short-video problematic use, learning engagement, and perceived learning ineffectiveness among Chinese vocational students. Healthcare 11:161. doi: 10.3390/healthcare11020161, PMID: 36673529 PMC9858663

[ref56] Ye J.-H. Wu Y.-T. Wu Y.-F. Chen M.-Y. Ye J.-N. (2022). Effects of short video addiction on the motivation and well-being of Chinese vocational college students. Front. Public Health 10:847672. doi: 10.3389/fpubh.2022.847672, PMID: 35619803 PMC9127725

[ref57] Ye J.-H. Zheng J. Nong W. Yang X. (2025b). Potential effect of short video usage intensity on short video addiction, perceived mood enhancement ('TikTok brain'), and attention control among Chinese adolescents. Int. J. Ment. Health Promot. 27, 271–286. doi: 10.32604/ijmhp.2025.059929

[ref58] Yu H. Mu Q. (2024). Effects of physical exercise on internet addiction among college students: mediated by stress, moderated by self-control. Soc. Behav. Pers. 52, 1–11. doi: 10.2224/sbp.13178

[ref59] Zhou J. Wang L. (2022). Differences in the effects of reading and aerobic exercise interventions on inhibitory control of college students with Mobile phone addiction. Front. Psych. 13:797780. doi: 10.3389/fpsyt.2022.797780, PMID: 35299822 PMC8920989

[ref60] Zhu H. Liu Y. Chen W. (2010). A study on the relationship between middle school students' attachment complex and social anxiety. Teach Adimist 10, 34–35.

